# The combined application of bleomycin with triamcinolone acetonide in port wine stains: inhibiting proliferation and recurrence of port wine stains

**DOI:** 10.1186/s40902-023-00395-5

**Published:** 2023-08-25

**Authors:** Quan-Feng Luo

**Affiliations:** Department of Oral and Maxillofacial Surgery, Peking University School and Hospital of Stomatology, Beijing, 100081 China

**Keywords:** Port wine stains, Bleomycin, Triamcinolone acetonide, Hypertrophy, Recurrence

## Abstract

**Background:**

Port wine stains slowly grow thicker over time, nodules appear on the surface, and the color slowly deepens from pink to purple. Even after laser treatment, some port wine stains will recur and slowly grow, and the erythema appears again. The purpose of this study was to investigate the effectiveness of bleomycin in combination with triamcinolone acetonide in the treatment of inhibiting the proliferation and recurrence of port wine stains.

**Results:**

Histopathological change: Before treatment, dense capillaries were distributed within the lesion, and blood fills the lumen. Lack of normal skin structure: After bleomycin and triamcinolone acetonide injection, the number of capillaries was significantly reduced, and fibrosis occurred in tissues. Changes in facial morphology: After treatment, the port wine stains became thinner, the asymmetry of the face was effectively improved, and the appearance have been significantly improved. After 5 years of follow-up, there were no recurrent cases.

**Conclusion:**

Bleomycin and triamcinolone acetonide injection can effectively inhibit the proliferation of port wine stains and prevent port wine stains from recurring after treatment.

## Background

Most of the port wine stains will proliferate and thicken over time, form nodules on the surface of the lesion, and distort the tissue morphology. Even after laser treatment or surgery, there is still a tendency to hyperplasia and hypertrophy. In particular, port wine stains involving deeper tissues or with syndrome are more prone to hyperplasia. To address this, we used bleomycin and triamcinolone acetonide for topical injection treatment of port wine stains. After the end of treatment, follow-up was carried out to observe the efficacy. The results showed that the combined application of bleomycin and triamcinolone acetonide could effectively inhibit the proliferation of port wine stains and thin the thickened lesion tissue. It is summarized below.

## Methods

### Patient’s information

From June 2011 to June 2021, patients with port wine stain and Sturge-Weber syndrome who have already developed significant hyperplasia and obvious asymmetric deformities in maxillofacial region were selected. There were 36 patients with port wine stains, 16 males and 20 females. Age ranged from 8 to 65 years. Among them, 11 patients were with Sturge-Weber syndrome, 4 men and 7 women. This retrospective study was approved by the bioethics committee of the School and Hospital of Stomatology. All patients signed an informed consent form prior to treatment and agreed to use clinical data for academic communication and publication.

### Medications

Bleomycin is produced by Pfizer Pharmaceutical Co., Ltd., 15,000 units/stick. Prepared with normal saline, the concentration of bleomycin is 500 units/ml. Triamcinolone acetonide is produced by Zhejiang Xianju Pharmaceutical Factory, 50 mg/5 ml. Prepared with normal saline, triamcinolone acetonide concentration is 5 mg/ml.

### Therapeutic method

Inject the drug into lesions with a 5-ml syringe. The injection volume was determined from age and the lesion surface area. Within the safe dose range of the drug applicable in the corresponding age group, the drug dose is determined according to the size of the lesion. The dosage of bleomycin was 500 units/cm^2^, and the dosage of triamcinolone acetonide was 5 mg/cm^2^. Drug injection depth: From the dermis layer to the subcutaneous fat layer, the bone attachment site was injected into the periosteum. Drugs were injected in manner of radiation dispersion, the injection spacing was less than 0.5 cm, and the drugs were evenly distributed in the lesions as much as possible. The interval between treatments was determined according to the hyperplasia status and size of port wine stains. If the area of port wine stains was large and the hyperplasia was obvious, it was difficult to cover all lesions with one administration, and the interval between administrations was 2 weeks. If the area of port wine stains was small, a single injection treatment can cover all lesions, and the interval was 4 weeks. For large areas of port wine stains, it can be taken for 2–3 consecutive days to cover all the lesions; the next injection was given after 3–4 weeks. Generally, the interval between initial treatment was short, and the interval between later periods was long. When port wine stains appeared atrophied and thinned, the interval between administrations was 2–3 months. The interval between administrations was not fixed and depended on the condition after each treatment. The lesion was usually injected with the drug 10–15 times. The use of triamcinolone acetonide depended on lesion hyperplasia.

### Clinical indication for discontinuation of treatment

1. When the volume of the lesion became significantly smaller, the tissue became thinner, and the affected side was close to symmetrical with the unaffected side; the treatment was stopped.
2. The surface of the lesion was wrinkled, and the erythema turned purple or subsided.

### Evaluation index

After the treatment, efficacy evaluation was performed, and patients were regularly reviewed and followed up for 4–5 years. Observing items are listed as follows.

### Change of histopathology

The histopathological changes of bright erythematous nevus before and after bleomycin treatment were compared.

### Changes in the thickness of the lesion

B-scan ultrasonography examination was performed on the same lesion location before and after treatment to observe the change of lesion thickness. Three positions were taken for each lesion, and the location sites were recorded. The measured data was analyzed by *t*-test.

### Changes in facial morphology

Before and after treatment, the lesion site and the face were photographed to observe the changes of lesion thickness, lesion surface color, and facial asymmetry. Patients without pulsed dye laser and photodynamic therapy were included in the observation of erythema change, and there were 7 patients. Patients who had undergone laser treatment were excluded. The plastic surgeon scored the lesions and facial morphology before and after the treatment. According to hyperplasia, facial asymmetry, and erythema, it was divided into five grades: (1) slight, (2) mild, (3) moderate, (4) sub-severe, and (5) severe. The obtained data was analyzed separately. Means and standard deviations were calculated. The Wilcoxon test (IBM SPSS Statistics 19 version) was used to evaluate the differences between the thickness, symmetry, and erythema before and after treatment. *P*-value < 0.05 was considered significant.

### Recurrence of the lesion

Observe whether hypertrophy and erythema exacerbate again. The change was divided into three grades: (1) no recurrence; (2) mild recurrence, slight hyperplasia, and slight color change; and (3) recurrence, obvious hyperplasia, and erythema darkening. *P*-value < 0.05 was considered significant.

### The patient’s self-evaluation

The change before and after treatment of the lesion was divided into three levels: (1) no change, (2) change, and (3) obvious change.

### Patient satisfaction with the results of treatment

The patient’s satisfaction with the treatment results was divided into three levels: (1) dissatisfied, (2) satisfied, and (3) very satisfied.

## Results

### Change of histopathology

**Fig. 1:**
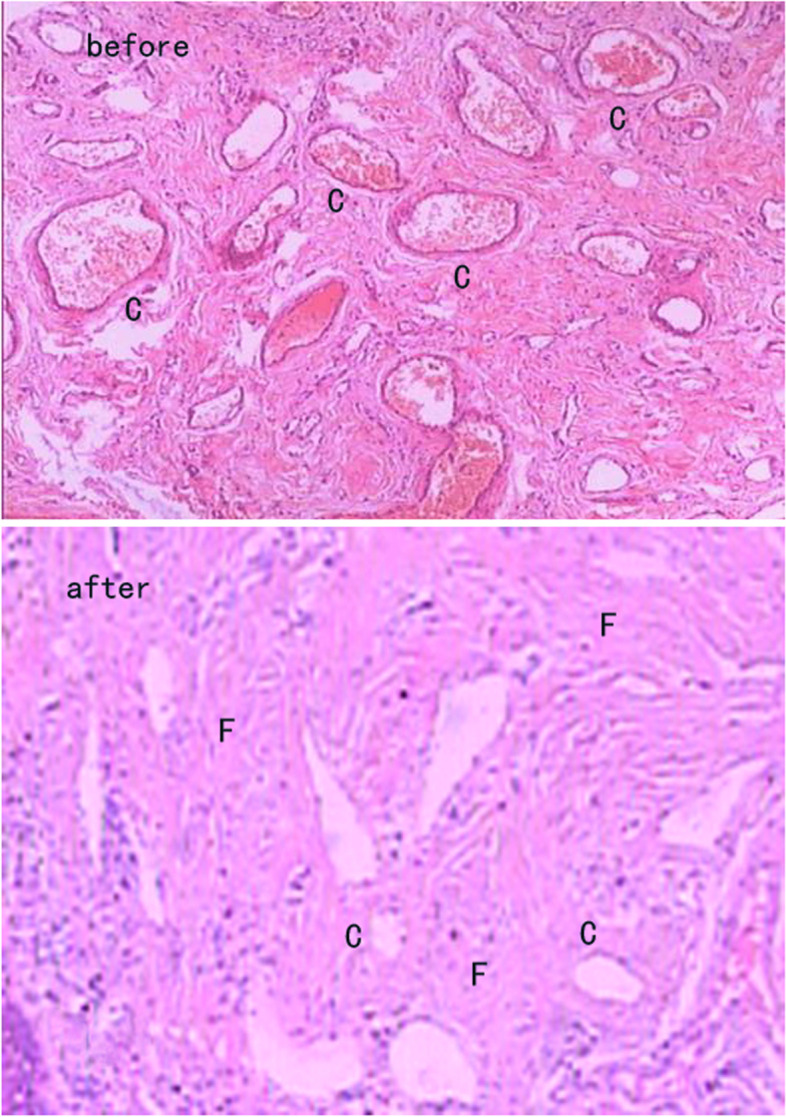
Histopathological change of PWS before and after treatment. Before treatment, a lot of capillaries were distributed within the lesions, and blood fills the lumen. Blood flow was abundant. Lack of normal skin structure, tissue structure was disordered. After bleomycin and triamcinolone acetonide injection, the number of capillaries was significantly reduced, blood flow in the lumens was reduced, and fibrosis occurred in tissues

Before treatment, dense capillaries are distributed within the lesion, and blood fills the lumen. Lack of normal skin structure, tissue structure is disordered. After bleomycin and triamcinolone acetonide injection, the number of capillaries is significantly reduced, blood flow in the lumens is reduced, and fibrosis occurs in tissues (Fig. 1).

### Changes in the thickness of the lesion

The statistical analysis of the lesion thickness showed that *P* < 0.05; there was a significant difference in the thickness of the lesion before and after treatment. It indicated that bleomycin and triamcinolone acetonide injection could reduce the thickness of the lesion.

B-scan ultrasonography examination showed that the subcutaneous tissue deep in the lesion also had hyperplasia, and the subcutaneous tissue thinned after bleomycin and triamcinolone acetonide treatment. But the changes in subcutaneous tissue were not included in the scope of the study, although the thinning of the subcutaneous tissue played an important role in correcting asymmetric deformities of the face (Table 1).

**Table 1:** Morphological changes of port wine stains evaluated by plastic surgeons and measurements by B-scan ultrasonography

	Evaluation by plastic surgeon			Lesion thickness (by B-scan ultrasonography)
	Thickness	Symmetry	Erythema	
Pre-treatment	3.92 ± 0.84	3.25 ± 0.81	3.71 ± 0.49	2.72 ± 0.46 mm
Post-treatment	2.47 ± 0.56	2.28 ± 0.51	3.43 ± 0.53	1.96 ± 0.36 mm
*P*	< 0.05	< 0.05	> 0.05	< 0.05

Statistical analysis of the scoring results showed that there were significant differences in the thickness of lesions and the symmetry of the face after the treatment of bleomycin and triamcinolone acetonide injection. There was no significant difference in the color of lesion erythema. The statistical analysis of the lesion thickness data obtained by B-scan ultrasonography examination before and after treatment showed that *P* < 0.05; there was a significant difference in the thickness of the lesion before and after treatment

### Changes in facial morphology

**Fig. 2:**
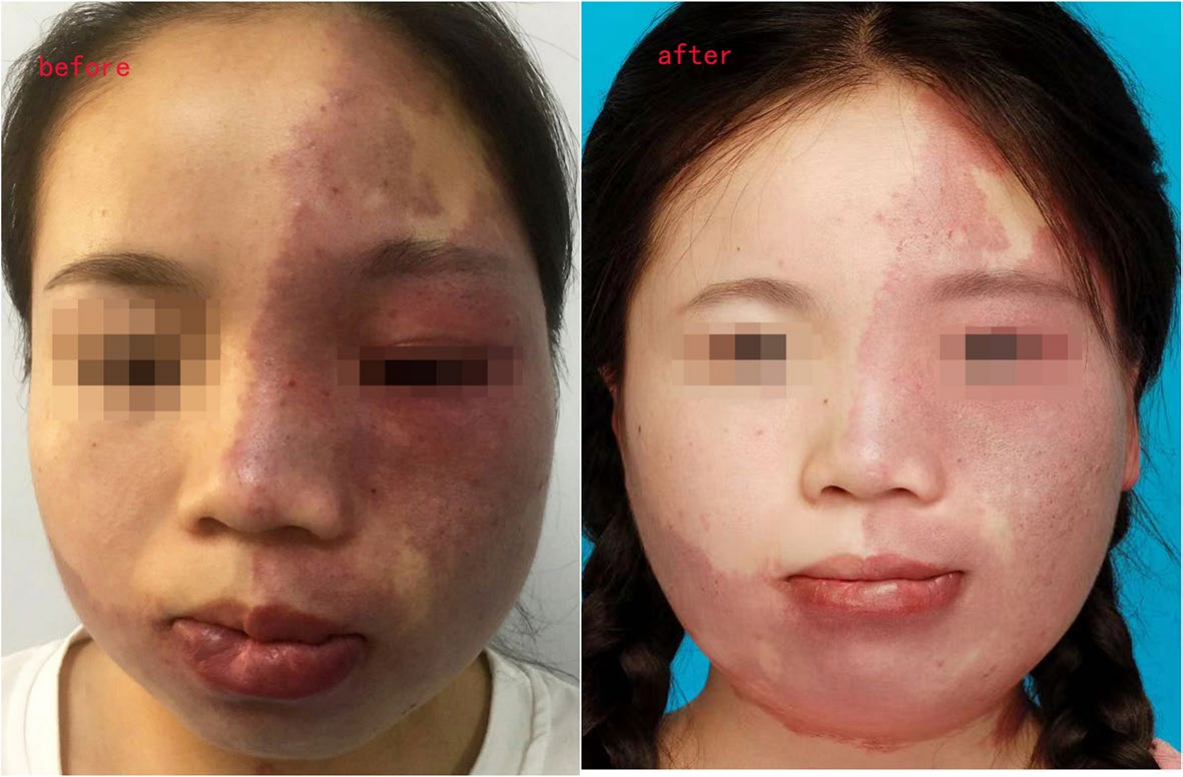
Changes of facial morphology in patients with Sturge-Weber syndrome. Female, 26 years old, with Sturge-Weber syndrome, hypertrophy on the left side of the face, severe asymmetric deformity. At the age of 14, she began to receive treatment with bleomycin and triamcinolone acetonide. After more than 3 years, the hypertrophy on the left face slowly subsided. The face returned to approximately symmetry. After 6 years of follow-up, the facial condition was stable, and no recurrence occurred

**Fig. 3:**
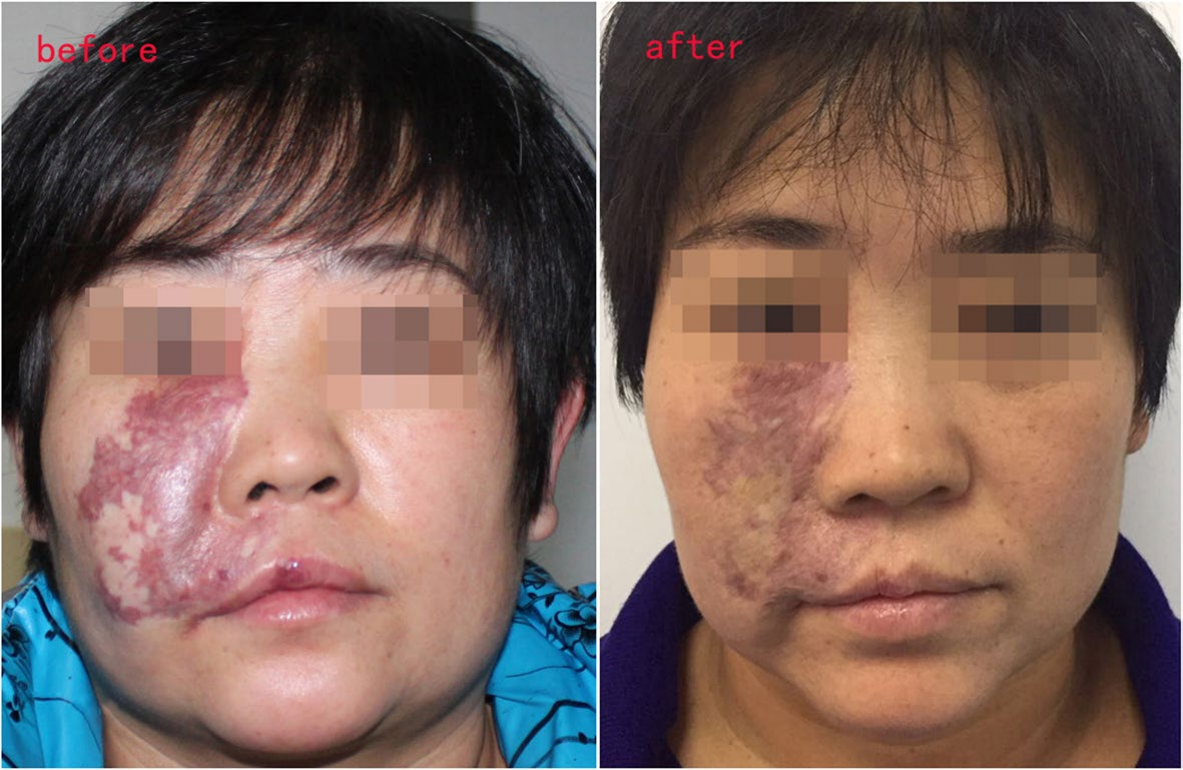
Changes of facial morphology in patients with port wine stains. Female, 46 years old, port wine stains on the right side of the face. At the age of 37, she began to receive injectable treatment with bleomycin and triamcinolone acetonide. After 3 years of treatment, the hypertrophy on the right side of the face slowly subsided. After 5 years of follow-up, the facial condition was stable, and no recurrence occurred

**Fig. 4:**
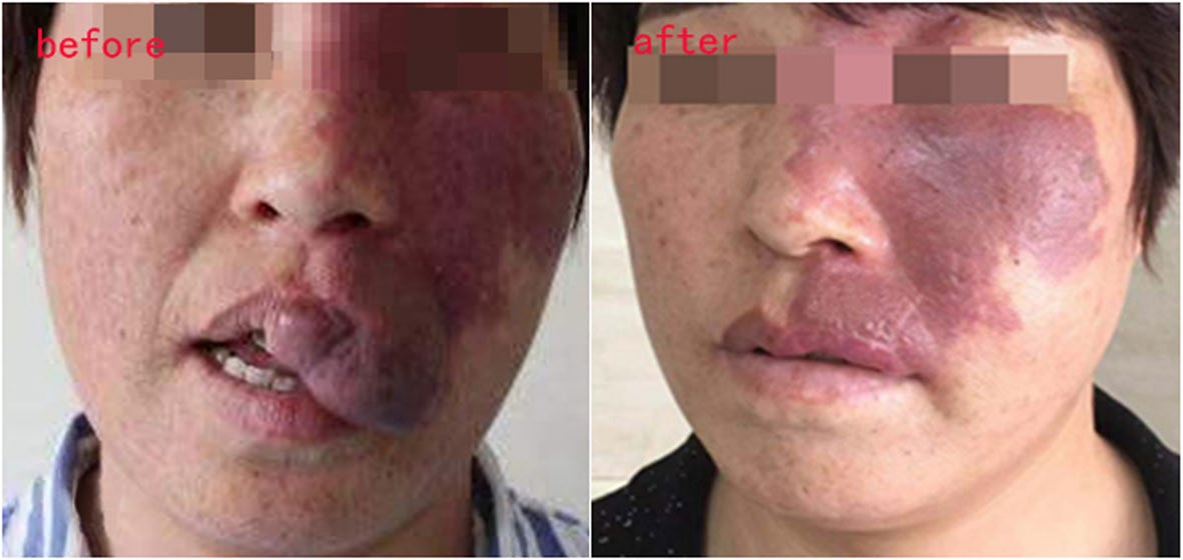
Changes of facial morphology in patients with port wine stains. Female, 43 years old, port wine stains on the left side of the face. Hyperplasia of the left upper lip and cheek, excision of part of the hypertrophic tissue, and then topical administration with bleomycin and triamcinolone acetonide. After 5 years of follow-up, the facial condition was stable, and no recurrence occurred

**Fig. 5:**
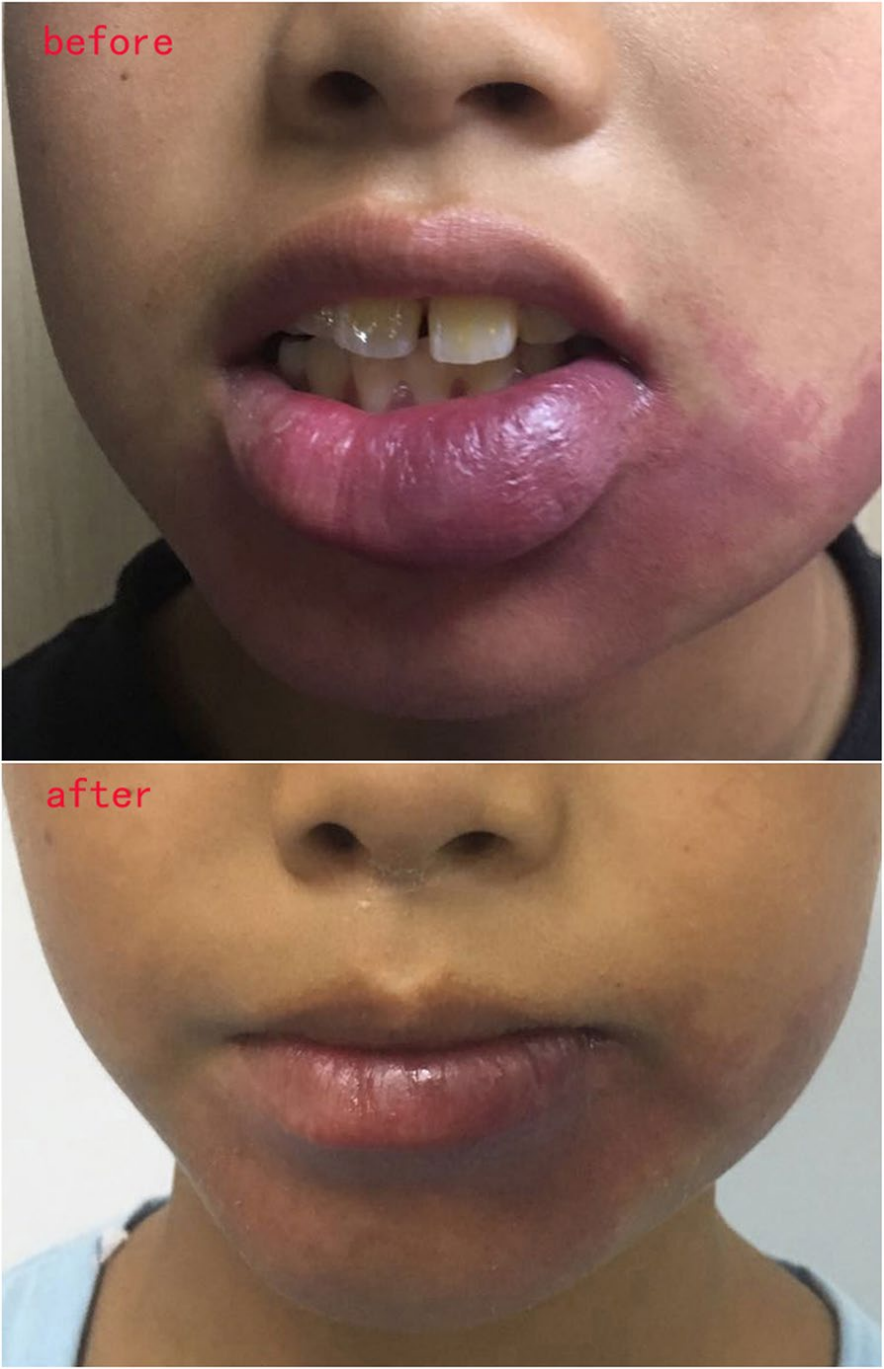
Changes of facial morphology in patients with port wine stains. Male child, 8 years old, port wine stains on lower lip and chin. After plastic surgery on the lower lip, topical administration was given with bleomycin and triamcinolone acetonide. After 4 years of follow-up, there is no hyperplasia on the lower lip and chin

Statistical analysis of the scoring results showed that there were significant differences in the thickness of lesions and the symmetry of the face after the treatment. There was no significant difference in lesion erythema. After injection treatment, the hypertrophic lesion on the affected side became thinner. Compared with the healthy side, the degree of bilateral asymmetry of the face has been significantly improved, and some patients have almost returned to symmetry. The lesion erythema regressed to varying degrees, the original bright color became dull, blood-filled lesions became wrinkled, but statistical analysis showed no significant difference between the erythema before and after treatment (Figs. 2, 3, 4, and 5, Table 1).

### Recurrence of the lesion

Among the patients who followed up, no recurrence was observed in those who completed all treatment. The patient who did not complete the treatment had a recurrence after stopping the treatment 10 months, 1 case, a girl, 12 years old, and with port wine stains on the chin and lips. The lower lip thickens again, and the erythema darkens. Bleomycin and triamcinolone acetonide were injected again to complete the full injection course. No recurrence of lesions was observed in subsequent observations (Table 2).

**Table 2:** The recurrence of port wine stains after laser therapy, patient’s self-evaluation of the effect, and satisfaction with the treatment

	Recurrence			Self-evaluation			Satisfaction		
PWS	1	2	3	1	2	3	1	2	3
	0	0	1	0	21	15	2	24	10

One case recurred in a girl of 12 years old with PWS on the chin and lips. All patients believed that there has been a change, and their symptoms have improved significantly. Two patients expressed dissatisfaction with the treatment effect. The remaining 34 patients were satisfied or very satisfied with the outcome of the treatment

### The patient’s self-evaluation

After treatment, patients believed that there has been a change, and their symptoms have improved significantly. Although one case relapsed, it was much better than the original lesion (Table 2).

### Patient satisfaction with the results of treatment

Two patients expressed dissatisfaction with the treatment effect and thought that the treatment did not achieve the desired effect. The remaining 34 patients were satisfied or very satisfied with the outcome of the treatment (Table 2).

## Discussion

Port wine stains usually undergo a slow process of development and change, the color changes from pink to purple, the lesions slowly thickens and bulges, and nodules gradually appear on the lesion surface [1–3]. The deformity of port wine stains is usually treated with laser and surgical excision [4–7]. Laser treatment can remove the nodules, but is not ideal for diffuse thickening. Surgical excision mainly excises dilated skin, and is not effective for making lesion thinner.

Erythema is mainly treated with pulsed dye lasers and/or photodynamic therapy. But some erythema is difficult to fade although having been treated with laser. Recurrence, hyperplasia, and erythema reappeared in some port wine stains [8–12].

Bleomycin is an antitumor drug, which binds to the DNA of the cell, causing the DNA strand to break, thereby blocking the division and proliferation of the cell; M-phase cells are most sensitive to bleomycin [13, 14]. In vitro experiments have shown that bleomycin can significantly disrupt the mitosis of fibroblasts [15]. We use bleomycin clinically for the treatment of diseases such as hemangioma and vascular malformations [16–18]. Bleomycin can effectively inhibit the proliferation of hemangiomas and make hemangiomas regress and resolve the venous malformation and vascular-lymphatic malformation.

We applied bleomycin in combination with triamcinolone acetonide to hypertrophic port wine stains and the recurrence after treatment. Bleomycin destroyed the proliferating capillaries and makes it fibrotic. Triamcinolone acetonide reduced collagen and mucopolysaccharide synthesis and accelerated collagen degradation and lysis of fibroblasts [19]. Triamcinolone acetonide can constrict blood vessels and reduce blood supply to tissues, resulting in tissue atrophy [20]. The combined application of bleomycin with triamcinolone acetonide exerted an inhibitory and degradation effect. Therefore, hyperplastic PWS thinned and no longer relapsed.

## Conclusion

Bleomycin and triamcinolone acetonide can effectively inhibit the proliferation of port wine stains and thin the hyperplastic lesion tissue. At the same time, it can effectively prevent the recurrence of port wine stains after treatment.

## Data Availability

All data generated or analyzed during this study are included in this article. Further enquiries can be directed to the corresponding author.
